# Low Neutralization of SARS‐CoV‐2 Omicron BA5248, XBB15 and JN1 by Homologous Booster and Breakthrough Infection

**DOI:** 10.1002/jmv.70189

**Published:** 2025-01-27

**Authors:** Jianhua Li, Hao Yan, Jiaxuan Li, Feng Ling, Yan Feng, Haiyan Mao, Xingxing Wang, Xiaoyan Li, Wanchen Song, Guangshang Wu, Yanjun Zhang, Yin Chen, Keda Chen

**Affiliations:** ^1^ Zhejiang Key Laboratory of Public Health Detection and Pathogenesis Research, Department of Microbiology Zhejiang Provincial Center for Disease Control and Prevention Hangzhou Zhejiang China; ^2^ Key Laboratory of Artificial Organs and Computational Medicine in Zhejiang Province, Shulan International Medical College Zhejiang Shuren University Hangzhou Zhejiang P. R. China; ^3^ Department of Infectious Diseases Zhejiang Provincial Center for Disease Control and Prevention Hangzhou Zhejiang China; ^4^ School of Medical Technology and Information Engineering Zhejiang Chinese Medical University Hangzhou Zhejiang China

**Keywords:** booster vaccination, breakthrough infections, COVID‐19, SARS‐CoV‐2

## Abstract

Immunity against Severe Acute Respiratory Syndrome Coronavirus 2 (SARS‐CoV‐2) can be induced through either infection with the virus or vaccination, providing protection against reinfection or reducing the risk of severe clinical outcomes. In this study, we recruited 172 volunteers who received different vaccination regimens, including 124 individuals who had recovered from breakthrough infections caused by the Omicron variant (27 with 2 doses, 49 with 3 doses, and 48 with 4 doses) and 48 healthy donors who did not experience breakthrough infections (all of whom received a fourth dose during the infection wave). We measured neutralizing antibody levels against Omicron BA.5.2.48, XBB.1.5, and JN.1 and found no significant differences in neutralizing antibody titers between natural infection and homologous booster vaccination at 6 months (*p* > 0.05), with geometric mean titers declining by over 100‐fold for some variants relative to the prototype strain.

## 1 Introduction

Vaccination against SARS‐CoV‐2 has been crucial in saving millions of lives that were at risk of severe COVID‐19. According to mathematical models, COVID‐19 vaccination can reduce mortality by 60% within the first year, though this rate varies with vaccine coverage. It is estimated that 14.4 million deaths could be prevented worldwide [1]. However, as new variants with significant immune escape capabilities continue to arise, these variants have largely evaded the immune defenses built through vaccination and natural infection.

Immunity against SARS‐CoV‐2 is induced either through infection with the virus or vaccination, providing protection against reinfection or reducing the risk of severe clinical outcomes [2]. A large‐scale study estimates that the protection rate of convalescent serum against SARS‐CoV‐2 reinfection is around 90%, whereas vaccine efficacy has been reported to range between 50% and 95% [3, 4]. However, the humoral response of memory B cells against SARS‐CoV‐2 diminishes over time [5, 6]. This raises significant concerns about the waning immunity in vaccinated individuals and those who have recovered from SARS‐CoV‐2 [7]. Additionally, reports indicate that the decline in SARS‐CoV‐2 antibody levels exhibits specificity, antibody titers in vaccinated individuals decrease by approximately 38% per month, compared to a monthly decrease of about 12% in previously infected but unvaccinated individuals [8].

In order to assess whether the number of vaccine doses influences antibody levels following breakthrough infections and to compare the neutralizing antibody activity of booster vaccines against natural pathogen exposure for SARS‐CoV‐2 and its variants, we analyzed neutralizing antibody levels against Omicron BA.5.2.48, Omicron XBB.1.5, and Omicron JN.1 in individuals who received two, three, or four doses of the vaccine and experienced breakthrough infections, as well as those who remained uninfected. These levels were then compared to those against the prototype.

## 2 Materials and Methods

### 2.1 Ethics Statements

The study was approved by the Chinese Centre for Disease Control and Prevention Institutional Review Board (202306).

### 2.2 Cell Culture

Vero cells (ATCC cc‐81; Sinovac Biotechnology, Beijing, China) were grown in a minimal essential medium (Gibco, Grand Island, NY, USA). BHK‐21‐hACE2 cells stably expressing Vero E6 (ATCC CRL‐1586) and human angiotensin‐converting enzyme 2 (ACE2) were provided by Professor Qin Xiaofeng (Center of Systems Medicine, Chinese Academy of Medical Sciences, Beijing, China). Both cell lines were grown in high glucose Dulbecco's modified Eagle's medium (Gibco). All media were supplemented with 10% fetal bovine serum (Gibco), 1% penicillin‐streptomycin, and 25 mM HEPES. Vero cell culture medium was supplemented with 2 mM l‐glutamine, and all cells were treated with EDTA (Gibco) containing 0.25% trypsin every 2–3 days.

### 2.3 Virus Stocks

Virus strains of SARS‐CoV‐2 provided by the Zhejiang Provincial Center for Disease Control and Prevention, Hangzhou, China, were used in our experiments. The following strains were isolated from throat swabs and cultured in Vero cells: SARS‐CoV‐2/Vero/WGF/2020/WZ122 (Prototype/EPI_ISL_12040150), SARS‐CoV‐2/E6/FJH/2022/ZJ104 (Omicron/BA.5.2), SARS‐CoV‐2/E6/YF/2023/ZJ78 (Omicron/XBB.1.5) and SARS‐CoV‐2/E6/HYH/2024/ZJ01 (Omicron/JN.1.5). A microdose cytopathic effect (CPE) assay was used to determine viral titers after harvesting cells at 50% CPE [9].

### 2.4 Blood Samples

Between May 3, 2023 and May 14, 2023, the Zhejiang Provincial Center for Disease Control and Prevention recruited 172 volunteers who had received different vaccination regimens of PiCoVacc (an inactivated viral vaccine developed by Sinovac Biotech). Among them were 124 individuals who had recovered from breakthrough infections caused by the Omicron variant after vaccination (27 received two doses, 49 received three doses, and 48 received four doses) and 48 healthy donors who did not experience breakthrough infections (all of whom received a fourth dose during the infection wave). Diagnosis of omicron infection post‐vaccination (OIP) was confirmed through real‐time reverse transcription polymerase chain reaction (RT‐PCR) testing of nasopharyngeal swab samples, with a positive result indicating OIP. Clinical parameters, including gender, age, vaccination history, and cycle threshold (Ct) values from nucleic acid tests, were extracted from medical records. Although we were unable to obtain viral samples for whole‐genome sequencing, data from SARS‐CoV‐2 variant surveillance indicated that B.1.1.529 variants accounted for most infections during that period.

### 2.5 Live Virus Neutralization Test

Serum samples were heat‐inactivated at 56°C for 30 min. The virus and diluted serum were mixed at a ratio of 1:1. In the CPE‐based assay, the authentic virus was neutralized in 96‐well plates with a viral titer of 10^10^ TCID50 (50% tissue culture infectious dose). The serum‐virus mixture was incubated at 37°C, 5% CO_2_ for 3 days after adding 1–2 × 10^4^ Vero E6 cells. The CPE was recorded microscopically for each well. It was determined that if 50% or more cells were protected from CPE at any dilution, and the reciprocal of that dilution was used to calculate the neutralization titer. The neutralization antibody titers were expressed as geometric mean titers. The live virus neutralizing antibodytest conducted in the biosafety level three laboratory of the Zhejiang Provincial Center for Disease Control and Prevention.

### 2.6 Pseudoviral Neutralization Assay

The vesicular stomatitis virus (VSV)‐based pseudovirus system was used to assess cross‐neutralizing activities in 172 convalescent serum specimens. The SARS‐CoV‐2/Prototype and SARS‐CoV‐2/Omicron pseudovirus systems were purchased from RayBiotech Life Inc. The sera were diluted, transferred into 96‐well culture plates, and then mixed with the SARS‐CoV‐2 pseudovirus (1 × 10^5^ TCID50/well).

After incubation for 1 h at 37°C, trypsinized BHK‐21‐hACE2 cells were added to the 96‐well culture plates with viruses/serum samples at a density of 2 × 10^4^/well. After 48 h, the number of GFP+ fluorescent cells were determined using a multi‐well plate imager (SparkCyto, Tecan, Männedorf, Switzerland). Then, the reciprocal of the dilution multiple corresponding to a 50% reduction in fluorescence value (IC50) compared to the negative control was designated as the neutralizing antibody (nAb) titer, using nonlinear regression curve fitting (normalized response, variable slope) in GraphPad 9.4.1 (GraphPad Inc., La Jolla, CA, USA). The neutralization antibody titers were expressed as geometric mean titers.

### 2.7 Statistical Analysis

Statistical analysis was performed using GraphPad Prism 9.4.1. Statistical significance was determined using the Mann–Whitney two‐sided U test, with *p* < 0.05 being considered significant.

## 3 Results

**Figure 1:**
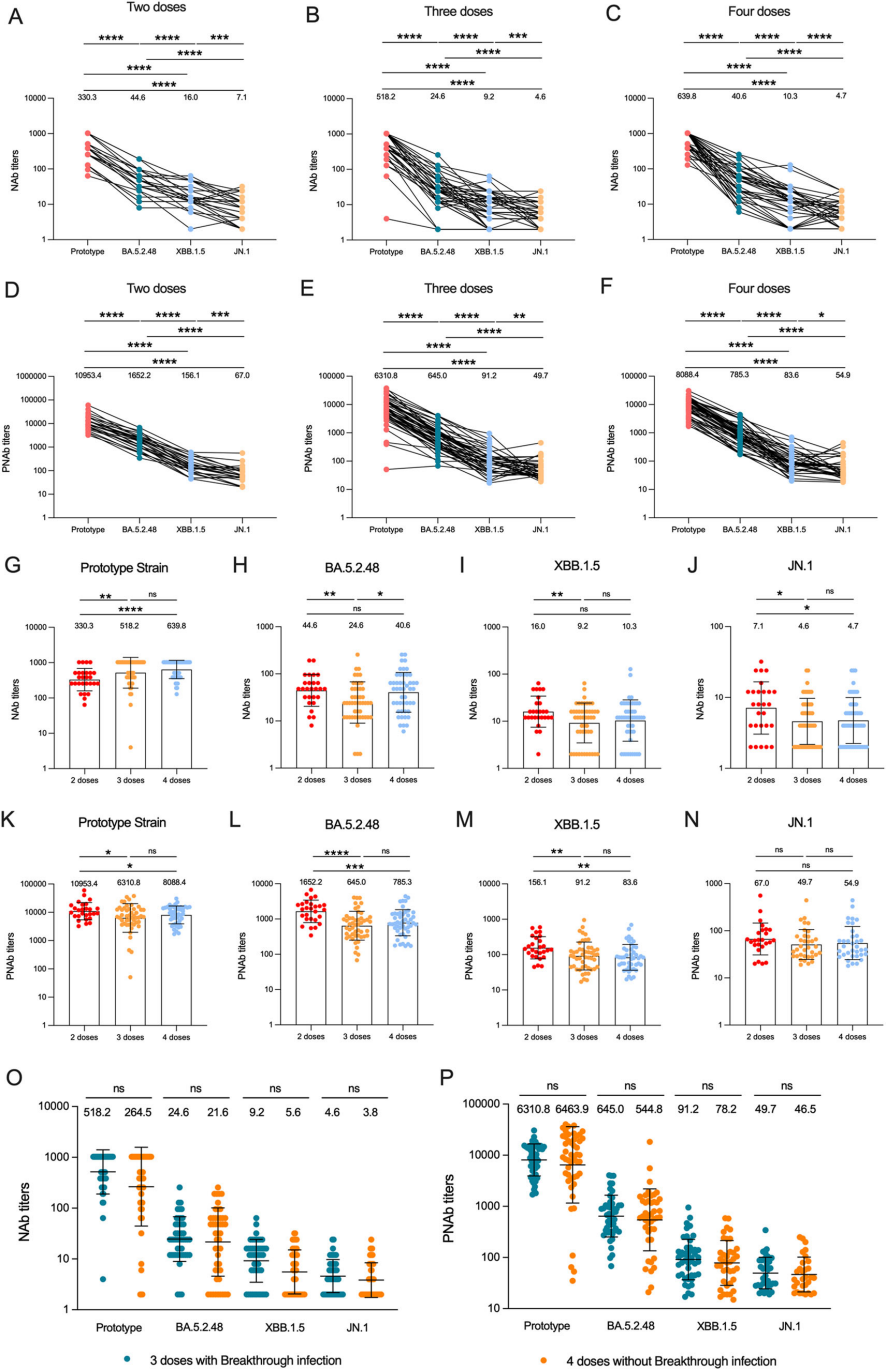
Comparison of antibody levels after different numbers of vaccinations and infections. (A–C) Comparison of live virus neutralization antibody levels against different strains in individuals who recovered from breakthrough infections after receiving 2 (A), 3 (B), or 4 (C) doses of inactivated vaccines. (D–F) Pseudovirus neutralization antibody results. (G–J) Comparison of live virus neutralization antibody levels against the prototype strain (G), BA.5.2.48 (H), XBB.1.5 (I), and JN.1 (J) in individuals who recovered from breakthrough infections after different numbers of inactivated vaccine doses. (K–N) Pseudovirus neutralization antibody results. (O) Comparison of live virus neutralization antibody levels between individuals who recovered from breakthrough infections after receiving three doses of inactivated vaccine and healthy volunteers who received four doses but did not experience infection. (P) Pseudovirus neutralization antibody results. The numbers in the figure represent the geometric mean titers of neutralizing antibodies for the cohort. The Mann‐Whitney test statistical method was used.

To determine whether BA.5.2.48, XBB.1.5, and JN.1 exhibit greater resistance to serum antibodies, we first assessed the neutralization activity of serum from three different clinical cohorts against these new subvariants. The results are summarized in Figure 1. These cohorts included patients who experienced breakthrough infections after receiving two, three, or four doses of the homologous PiCoVacc. Relevant clinical information for these participants is provided in Table 1. Consistent with previous studies, neutralizing titers against BA.5.2.48, XBB.1.5, and JN.1 were significantly lower than those against the prototype across all three cohorts, with reductions even more than 136‐fold (Figure 1A–C). Notably, antibody levels against the corresponding breakthrough strain Omicron BA.5.2.48 were higher than those against XBB.1.5 and JN.1, possibly due to the enhanced serum neutralization escape capabilities of the subsequent variants. Additionally, the geometric mean titers against Omicron BA.5.2.48 were significantly reduced compared to the prototype, with declines ranging from 7.4‐fold to 21.1‐fold. Similar trends were observed in pseudovirus experiments (Figure 1D–F). Despite detecting titers against XBB.1.5 and JN.1 in all sera, the geometric mean titers were low, suggesting that breakthrough infections may offer limited protection against reinfection.

**Table 1:** Clinical characteristics of the study population.

Group	Vaccination status	N	Age (Mean ± SD)	Gender (M/F)
Breakthrough infections	2 doses	27	9.6	20/7
	3 doses	49	57.1	18/31
	4 doses	48	54.1	18/20
Uninfected	4 doses	48	53.9	11/27

We also compared the impact of different vaccination regimens on antibody levels following breakthrough infections. In live virus neutralization tests, results aligned with expectations, showing no statistically significant difference between those who received three and four doses (Figure 1G–J). However, antibody levels appeared higher in those who received three doses compared to those who received two. Interestingly, in pseudovirus neutralization tests, serum antibody levels in individuals who recovered from breakthrough infections after two doses were notably higher than those in individuals who received three or four doses, particularly against the corresponding Omicron BA.5.2.48 strain (Figure 1K–N). This finding may require further investigation with larger sample sizes for validation and explanation.

To assess the neutralizing antibody activity induced by booster vaccination versus natural pathogen infection against SARS‐CoV‐2 and its variants, we compared individuals who experienced breakthrough infections after receiving three doses of the homologous PiCoVacc with those who received a second booster during the same period. Our results showed no statistically significant differences in antibody levels against prototype, BA.5.2.48, XBB.1.5, and JN.1 between those who received a booster and those who experienced natural breakthrough infections, suggesting that in these samples, antibody responses induced by SARS‐CoV‐2 breakthrough infections were not superior to those induced by vaccination (Figure 1O,P).

## 4 Discussion

Our results indicate that the Omicron variant, particularly JN.1, exhibits a strong capacity for immune evasion against immune defenses developed through vaccination and natural infection. Emerging subvariants may further compromise the effectiveness of current COVID‐19 vaccines, leading to an increase in breakthrough infections and reinfections. A large‐scale study comparing the rate of antibody decline after infection or vaccination found differing dynamics in the waning of B‐cell and T‐cell responses in individuals infected with SARS‐CoV‐2. In this study, unvaccinated individuals with prior infection (*n* = 4361) exhibited a slower decline in antibody titers compared to fully vaccinated individuals (*n* = 2653; two doses of BNT162b2), which may help explain the results observed in our pseudovirus neutralization assays [10]. However, larger sample sizes are needed to validate these findings. Consequently, further evaluation is required to determine the duration of protection, the necessity of additional doses, and whether Omicron‐adapted vaccines are needed.

When comparing different vaccination regimens, we observed that individuals receiving three doses generally exhibited higher antibody levels than those receiving two doses. However, the additional increase after the fourth dose was limited, suggesting that immune responses may reach saturation after multiple doses. Interestingly, although antibody levels in the three‐dose group were higher than in the two‐dose group, some individuals with only two doses and subsequent breakthrough infections exhibited unexpectedly high antibody levels. This finding highlights the potential advantage of hybrid immunity, where the combination of vaccination and natural infection may enhance cross‐protection against emerging variants. In contrast, the antibody levels in individuals receiving four doses were not significantly higher than those in the three‐dose group, indicating that the effect of additional doses may have an upper limit. Since the efficacy of both vaccines and infections wanes over time, further research is needed to optimize vaccination strategies and extend the duration of immune protection. Our data suggest that natural breakthrough infections do not result in higher antibody levels compared to homologous booster doses, possibly due to the waning efficacy of both vaccines and breakthrough infections over time, leading to minimal differences between the two. This underscores the potential necessity of additional doses to maintain efficacy against Omicron subvariants. Given the significant rise in vaccine breakthrough infections, it remains important to adhere to strict infection control guidelines, even after vaccination.

While NAbs are often used as a surrogate for immune protection, they are not definitive correlates of protection [11]. Comprehensive immune protection depends not only on antibody levels but also on cellular immunity, including T‐cell responses and memory B cells. T cells play a critical role in clearing viral infections and maintaining long‐term immune memory, while memory B cells help rapidly generate new antibodies upon future infections [12, 13]. Therefore, relying solely on neutralizing antibody levels to assess vaccine efficacy may overlook other essential immune mechanisms. Future studies should integrate analyses of both neutralizing antibodies and cellular immune responses to comprehensively evaluate vaccine protection [14].

This study has several limitations. First, the cross‐sectional design restricts our ability to monitor dynamic changes in antibody levels and immune responses over time. This limits our understanding of antibody kinetics and the duration of immunity following vaccination or infection. Future studies should adopt longitudinal designs to better track these changes. Second, the sample size was relatively small, and demographic mismatches among different vaccination groups may have introduced variability. For example, differences in time intervals between vaccination, infection, and serum sampling may have affected antibody levels. Additionally, as serum samples were collected 6 months after breakthrough infections, they may not accurately reflect early changes in antibody levels post‐infection or vaccination. Third, this study did not assess T‐cell immunity or memory B cell responses, which are critical for understanding comprehensive immune protection offered by hybrid immunity. Future research should further investigate the role of these cellular components, particularly their contribution to immune responses after vaccination and infection. Finally, as Omicron and its subvariants continue to evolve, it is unclear whether the protection offered by current vaccines can be sustained over time. Thus, future research should not only expand sample sizes but also evaluate the effectiveness of Omicron‐specific vaccines. It is essential to determine whether additional booster doses are necessary and whether regular vaccination will be required to counter emerging immune‐escape variants.

## Author Contributions

Conceptualization, Yanjun Zhang, Haiyan Mao and Keda Chen; Methodology, J.L., and Hao Yan; Validation, Feng Ling, Yan Feng, Yin Chen, and Xiaoyan Li; Formal analysis, J.L., Wanchen Song, and Guangshang Wu; investigation, J.L., Xingxing Wang, and Keda Chen; resources, Yanjun Zhang, Haiyan Mao, and K.D.; writing–review and editing, J.L., J.L., and Keda Chen; supervision, Yanjun Zhang, Haiyan Mao, and Keda Chen; project funding acquisition, Yanjun Zhang, Haiyan Mao, and Keda Chen. All authors have read and agreed to the published version of the manuscript.

## Ethics Statement

The study was approved by the Chinese Centre for Disease Control and Prevention Institutional Review Board.

## Consent

The authors have nothing to report.

## Conflicts of Interest

The authors declare no conflicts of interest.

## Data Availability

The data that support the findings of this study are available on request from the corresponding author. The data are not publicly available due to privacy or ethical restrictions. The data presented in this study are available on request from the corresponding author.
